# Trends and Inequalities in Maternal and Newborn Health Services for Unplanned Settlements of Lusaka City, Zambia

**DOI:** 10.1007/s11524-024-00837-z

**Published:** 2024-03-08

**Authors:** Choolwe Jacobs, Mwiche Musukuma, Raymond Hamoonga, Brivine Sikapande, Ovost Chooye, Fernando C. Wehrmeister, Charles Michelo, Andrea K. Blanchard

**Affiliations:** 1 Department of Epidemiology and Biostatistics, School of Public Health, University of Zambia, Lusaka, Zambia; 2 Zambia National Public Health Institute, Lusaka, Zambia; 3 Ministry of Health, Lusaka, Zambia; 4 International Center for Equity in Health, Federal University of Pelotas, Pelotas, Brazil; 5 Harvest University, Lusaka, Zambia; 6 Department of Community Health Sciences, University of Manitoba, Winnipeg, Canada

**Keywords:** Maternal and newborn health services, Antenatal care, Skilled birth attendance, Cesarean sections, Emergency obstetric care, Urban health, Unplanned settlements, Health inequalities

## Abstract

Living conditions and other factors in urban unplanned settlements present unique challenges for improving maternal and newborn health (MNH), yet MNH inequalities associated with such challenges are not well understood. This study examined trends and inequalities in coverage of MNH services in the last 20 years in unplanned and planned settlements of Lusaka City, Zambia. Geospatial information was used to map Lusaka’s settlements and health facilities. Zambia Demographic Health Surveys (ZDHS 2001, 2007, 2013/2014, and 2018) were used to compare antenatal care (ANC), institutional delivery, and Cesarean section (C-section) coverage, and neonatal mortality rates between the poorer 60% and richer 40% households. Health Management Information System (HMIS) data from 2018 to 2021 were used to compute service volumes and coverage rates for ANC1 and ANC4, and institutional delivery and C-sections by facility level and type in planned and unplanned settlements. Although the correlation is not exact, our data analysis showed close alignment; and thus, we opted to use the 60% poorer and 40% richer groups as a proxy for households in unplanned versus planned settlements. Unplanned settlements were serviced by primary centers or first-level hospitals. ZDHS findings show that by 2018, at least one ANC visit and institutional delivery became nearly universal throughout Lusaka, but early and four or more ANC visits, C-sections, and neonatal mortality rates remained worse among poorer than richer women in ZDHS. In HMIS, ANC and institutional delivery volumes were highest in public facilities, especially in unplanned settlements. The volume of C-sections was much greater within facilities in planned than unplanned settlements. Our study exposed persistent gaps in timing and use of ANC and emergency obstetric care between unplanned and planned communities. Closing such gaps requires strengthening outreach early and consistently in pregnancy and increasing emergency obstetric care capacities and referrals to improve access to important MNH services for women and newborns in Lusaka’s unplanned settlements.

**Supplementary Information:**

The online version contains supplementary material available at 10.1007/s11524-024-00837-z.

## Background

Urban areas generally have better access to health care services and basic needs such as improved water, sanitation, and other infrastructure than rural locales. Such advantages continue to fuel massive rural-to-urban migration, leading to a high rate of urbanization in many African countries [1]. The population of Zambia’s capital, Lusaka, rose almost tenfold in the last five decades, from 353,975 in 1969 to 3,360,183 by 2020 [2], a surge that has caused extensive housing shortages and contributed to the significant increase in unplanned and illegal settlements [3]. Currently, about 70% of Lusaka City’s population lives in unplanned settlements [4]. The term “unplanned settlements” refers to any uncoordinated settlement without predetermined planning standards and poor provision of basic services and sanitation, a situation that poses health risks to people living there [5, 6]. Substandard living conditions and other socioeconomic disadvantages in urban unplanned settlements present unique challenges for improving maternal and newborn health (MNH), yet these challenges have not been well understood to date [7].

The health and safety of a pregnant woman and her baby can be improved by providing affordable access to good quality services for antenatal, delivery, and postnatal care [8]. According to ZDHS 2018, coverage of MNH services for women and newborns in rural Zambia has caught up to urban areas, including Lusaka [9]. Studies show that despite generally good availability of antenatal care (ANC) services overall, not all women in Lusaka receive timely or quality ANC services [10]. Previous studies found that while most women in unplanned settlements of Lusaka now prefer institutional deliveries, many deliver from home due to unrecognized labor symptoms or barriers such as limited resources for transport to a health facility or to buy delivery packages required by such facilities [11, 12].

Neonatal mortality rates (NMRs) appear to have stagnated in urban areas of Zambia. In Lusaka, the NMR was estimated to have increased slightly from 24 to 29 deaths per 1000 live births between the 2013 and 2018 ZDHS, which is higher than in Zambia’s rural areas (23 deaths per 1000 live births as per the 2018 ZDHS) [9, 13]. Neonatal deaths have been linked to delays in receiving skilled delivery and timely emergency care through the referral system [12]. The maternal mortality ratio (MMR) in Zambia was estimated to have decreased from 398 per 100,000 live births in the 2013/2014 ZDHS to 278 per 100,000 in the 2018 ZDHS, but subnational estimates are not readily available [9, 13]. Maternal deaths have been attributed to delays in seeking obstetric services, poor case management, and lack of skilled personnel [14].

Studies describing the sociodemographic characteristics, available health care infrastructure, and population coverage of MNH services for women in unplanned settlements in Lusaka City are limited [15, 16]. This is the first study to examine trends and inequalities in utilization of MNH services and the NMR in the last 20 years in unplanned compared to planned settlements of the city. Specifically, our objectives are (1) to determine the location and population density of unplanned and planned settlements in Lusaka, and the distribution and types of health facilities providing MNH services; and (2) to understand the trends in MNH service utilization and neonatal outcomes in unplanned, compared to planned, settlements of Lusaka. To do so, we integrated multiple population, geospatial, and facility-based datasets.

## Methods

### Study Setting

In Zambia, most unplanned settlements are either near the city center, within the industrial areas, or on the outskirts along major roads [17, 18]. About 35 unplanned settlements have been approved by the Ministry of Health as improvement areas, but ensuring adequate resources and services remains an enormous challenge [18]. As a result, these areas are not sustainably provided with essential infrastructure for electricity, water, sanitation, and effective solid waste management [18–20].

### Data Sources

Multiple data sources were used, including geospatial information on location of unplanned and planned settlements and facilities; the 2017 Zambia National Health Facility Census (ZNHFC); Zambia Demographic Health Surveys (ZDHS 2001, 2007, 2013/2014, and 2018); and monthly Health Management Information System (HMIS) data from 2018 to 2021. The HMIS data are collected on a daily basis and recorded through routine registers. These data are aggregated at the end of every month and reported through the Health Information Aggregation (HIA) tools for entry into the District Health Information System (DHIS2) [21].

For objective 1, we mapped population densities in each settlement, and health facilities by level, using global positioning system (GPS) coordinates. We also used HMIS (2021) and ZNHFC (2017) data to enumerate facilities at each level providing MNH services within Lusaka. Data from earlier periods were not consistently available, so we focused on the current infrastructure.

For objective 2, first we analyzed intervention coverage trends and inequalities using the four ZDHS surveys within the Lusaka urban cluster. Second, we analyzed service volumes and rates using HMIS facility-level data by month. Data were aggregated from 2018 to 2021 to gain a picture of the current situation, which is comparable to the period of ZDHS 2018 (although HMIS provided volumes and not coverage estimates, as denominator data were not available).

### Definitions of Indicators

Coverage indicators analyzed in ZDHS included at least one or any ANC visits (ANC1), early ANC (before 4 weeks), having four or more ANC visits (ANC4), institutional delivery, and Cesarean section (C-section) rates. Postnatal care was assessed, but not included, because estimates were not consistent over time. We also analyzed neonatal and under-5 mortality rates, as explained more below. From the HMIS, the indicators we used were the volume of first ANC visits (ANC1), volume of fourth ANC visits (ANC4), institutional deliveries, and C-sections.

### ZDHS Data Analysis

Coverage rates were computed among births in the previous five years using Stata 16.0. Using the syncmrates command in Stata, we also computed neonatal mortality rates (and under-5 mortality rates for comparison with NMR patterns, given under-5 mortality provides larger samples) among births in the previous 10 years in ZDHS 2007 and 2018. These analyses were disaggregated between the poorer 60% and richer 40% of the population, using the scores from the original survey datasets but reorganized based on the distribution of household wealth scores in Lusaka urban clusters only. Wealth index scores were previously created using a principal component analysis of dwelling materials, access to utilities, and household assets [22]. Re-calculating the scores for this setting yields correlation coefficients with the original measure of wealth of above 0.95 in all surveys (data not shown). As this is a relative position of wealth, the score could thus sufficiently discriminate among wealth groups in this setting, even without recalculating the scores for surveyed households in Lusaka City.

The use of 60% poorer aligns with the proportion of the population in Lusaka City’s unplanned settlements of 70%. Because ZDHS cluster GIS coordinates are displaced by up to 2 km in any direction, we could not use them exclusively to determine whether each cluster was predominantly in an unplanned or planned settlement. Population density could also not be used to distinguish between settlement types due to construction of densely populated large apartment buildings in planned settlements. Instead, we overlaid the GIS coordinates of the ZDHS 2018 survey clusters with maps of unplanned settlements using QGIS. We identified a few ZDHS clusters that fell almost completely within the radius of unplanned settlements even after the 2-km coordinate displacement buffer, and a few that fell within the radius of planned settlements after displacement. Then, we found that the majority (75% or more) of households in the clusters falling within unplanned settlements had shared toilets and/or pit latrine or flush-to-pit latrine toilets. In contrast, few clusters that fell completely within the 2-km buffer for planned settlements had these toilet characteristics. Hence, we used a 75% threshold for the toilet indicator to categorize the rest of the survey clusters in Lusaka as unplanned or planned settlements.

We then compared the MNH coverage estimates among clusters classified as “unplanned” using those sanitation data with coverage among the 60% poorer households using the wealth index, and similar coverage among clusters classified as “planned” using sanitation data with coverage among the 40% richer households (merging individual and household data) (Supplementary Fig. 1). As these coverage values aligned well, we opted to use the 60% poorer and 40% richer groups as a proxy for households in unplanned versus planned settlements [23]. We applied this for all ZDHS rounds, as earlier rounds did not have adequate samples and related GIS coordinates. Still, the proportion of households sharing a toilet or with pit latrines in Lusaka was similar at around 75% overall, and 80% among the poorest in 2001 and 2007.

### HMIS Data Analysis

We used HMIS data for analysis for both objectives 1 and 2; we first addressed issues of limited reporting and incomplete data. We identified facilities with data that provided MNH services in urban Lusaka, and reviewed data completeness. Around 60% of public facilities and 35% of private facilities reported ANC1 data for at least 6 months of a given year. The proportion of facility-months with data for ANC1, among facilities providing the service, was 95% for public facilities and 82% for private facilities across years (2018–2021). We addressed missing facility-month data by imputing the mean value of the previous or following 6 months for that facility (whichever was most complete).

Using the adjusted data, we computed total volumes across 2018–2021 for one and four ANC visits, institutional delivery, and C-sections. We also computed ratios of ANC1 to ANC4, and ANC1 to institutional delivery to assess utilization patterns. We calculated institutional C-section rates as the proportion of people coming for a delivery who had a C-section among facilities where this service was provided. We compared these between public and private facilities by each facility level (primary level included urban health posts, health centers, and clinics; hospital level included first- to third-level hospitals and others). We also compared utilization data between facilities proximal to and serving unplanned settlements with facilities located in planned settlements.

## Results

**Fig. 1:**
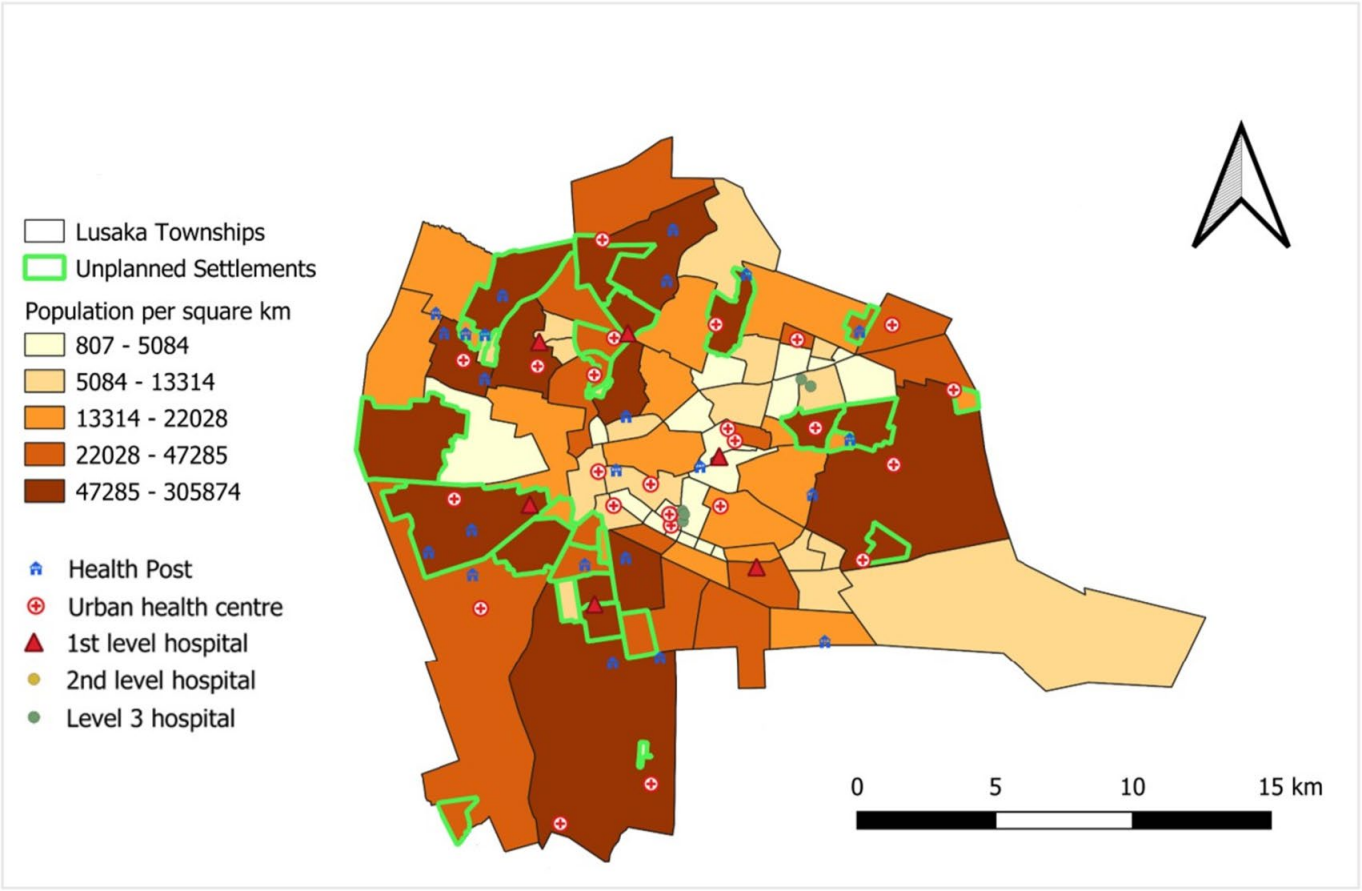
Map of Lusaka City with township population densities, location of unplanned settlements and health facilities by level

To answer the first objective, we used our data to create a map with unplanned settlement locations and population densities (Fig. 1). The majority of unplanned settlements are serviced by health posts or urban health centers (both primary level) and first-level hospitals. However, no second- or third-level hospitals are located in unplanned settlements.

Based on current HMIS records, approximately 164 health facilities exist in Lusaka, with about 77 government/public facilities and 87 private facilities. Just over 50% of the city’s public facilities, and 3% of its private ones, are located in unplanned settlements, with the rest in planned settlements. Both public and private facilities provide MNH services, although the share of government facilities providing such services is greater than the share of private facilities that offer them (see ZDHS results below). Of the private facilities that provide MNH services, just under two-thirds are primary centers or clinics (with varying capabilities), and over a third are hospitals.

**Fig. 2:**
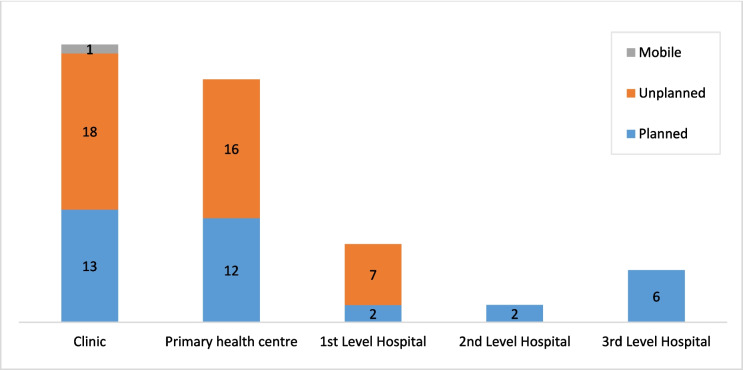
Number of public/government facilities (out of 77 total) by level and catchment area in Lusaka, HMIS 2021

Public health facilities in Lusaka can be categorized into five different types, including health posts, urban health centers, and first-, second-, and third-level hospitals. Lusaka has 32 health posts (also called clinics clinics) that are government run, 18 of which are in unplanned settlements (Fig. 2). These facilities offer promotional and preventive health services, each serving around 7000 or more people. About 36% of the 77 government-owned health facilities falling under the categorization of urban health centers (also termed “primary health centers”). These centers cater to catchment populations of between 30,000 and 50,000 people.

Nine of the 77 government-owned facilities are first-level (or district-level) hospitals. They are meant to serve around 400,000 people, though sometimes they serve more (78% of these are found in unplanned settlements). Second-level hospitals provide curative care and serve catchment populations of between 200,000 and 800,000 people. Only two government-owned health facilities are second-level hospitals. Tertiary or third-level hospitals provide the most specialized care. Lusaka has six government-owned third-level hospitals, with two offering advanced MNH services to the entire city.

The 2017 ZNHFC was conducted in 62 Lusaka public health facilities. It shows that ANC services (including hemoglobin tests, urine tests, and tetanus toxoid injections) were offered at most health posts and all urban health centers, first- and second-level hospitals, and at third-level hospitals. Institutional delivery was offered at a fifth of health centers, with those facilities providing basic emergency obstetric and newborn care (BEmONC) services, including injectable antibiotics, oxytocic drugs, manual removal of the placental and retained products of conception, assisted vaginal delivery, and neonatal resuscitation. Most first-, second-, and third-level public hospitals also conducted delivery services, with all reporting the ability to conduct BEmONC as well as C-sections and blood tranfusions (comprehensive emergency obstetric and newborn care, or CEmONC).

For referral pathways, urban health centers providing BEmONC refer to first-level hospitals for ANC and delivery care in cases of complications identified earlier during pregnancy (e.g., premature rupture of membrane, bleeding); after 34 weeks gestation (e.g., malpresentation, under age 16, contracted pelvis, fetal distress, obstructed labor); or postpartum (e.g., puerperal sepsis, postpartum hemorrhage) [24]. Second- and third-level hospitals receive referrals from urban health centers or first-level hospitals for specialized care, such as for women with pre-existing medical conditions, pre-eclampsia/eclampsia with other complications, placenta abnormalities, hemorrhage, or premature labor when detected. Newborns are also to be referred when adverse health conditions or delivery complications arise, from urban health centers to first-level hospitals, or first level to second and third levels depending on severity [24].

For the study’s second objective, we used ZDHS to compare trends and inequalities in MNH intervention coverage and mortality between women living in the poorer 60% (approximating those in unplanned settlements) and richer 40% (approximating those in planned settlements) of households in Lusaka from 2001 to 2018. Any ANC became nearly universal among pregnant women within Lusaka (over 97% for all areas in 2018). By 2018, the use of ANC was higher at public than private facilities (though slightly higher in the latter among the richer). Among the poorer 60%, about 74% received any ANC at a public health center and 21% at a public hospital, compared to 1% at a private hospital/clinic and 3% at a mission hospital/clinic. Among the richer 40%, about 56% received any ANC at a public health center, 30% at a public hospital, 7% at private hospital/clinic, and 2% at a mission hospital/clinic.

**Fig. 3:**
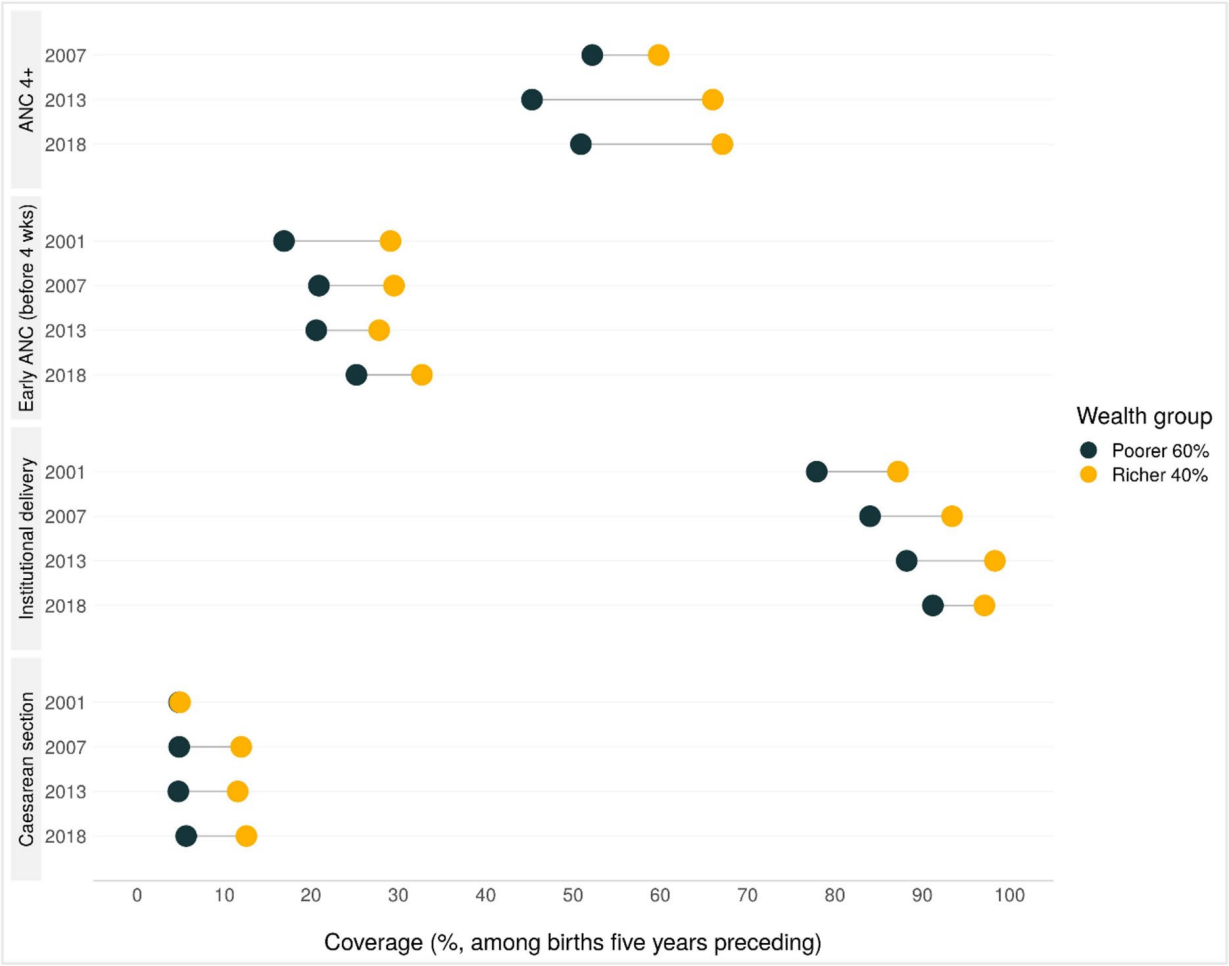
Intervention coverage for ANC, institutional delivery, and C-sections comparing women in the poorer 60% and richer 40% areas of Lusaka, ZDHS surveys 2001, 2007, 2013, 2018

Coverages of early and four or more ANC visits, institutional delivery, and C-sections among the poorer 60% and richer 40% are presented in Fig. 3. Estimates and confidence intervals are provided in Supplementary Table 1. Coverage of four or more ANC visits (ANC4) in both wealth classifications was substantially lower than any ANC, and improved for women residing in richer areas from 2007 to 2018. This caused the gap between poorer and richer to widen from 7.6 percentage points (pp) to 16.2 pp or a ratio of 1.2 to 1.3 between 2007 and 2018. Data in 2001 showed unrealistically high coverage (and was excluded), suggesting the way ANC4 was measured changed after that year. Early ANC (before 4 weeks gestation) increased somewhat among women from both poorer and richer areas. Inequalities reduced somewhat between the poorer 60% and richer 40%, from a rate difference of 12.3 to 7.5 points, or a ratio of 1.7 to 1.3, respectively between the 2001 and 2018 ZDHS. Still, coverage of early ANC remained low at below 35% for both groups.

Regarding institutional deliveries, higher coverage was observed among women from both poorer and richer areas between 2001 and 2018 ZDHS. Although institutional delivery coverage has been lower among women from poorer than richer areas, it rose slightly faster from ZDHS 2001 to 2018 among the poorer (78 to 91% respectively) compared to the richer (87 to 97%). Looking at type of facility where women gave birth in 2018 ZDHS, around 44% of those in the poorest 60% gave birth at a public health center and 42% at a public hospital (and less than 2% at a health post), versus 0.5% at a private hospital/clinic and 3% at a mission hospital/clinic. Among women from the 40% richer group in 2018, 33% delivered at a public health center, 56% at a public hospital, and 6% at a private hospital/clinic (and less than 1% at a mission hospital/clinic or government health post).

Coverage of C-sections was lower among women from the poorer than the richer areas across all survey periods. Rates started low at around 4.8% in both groups in 2001. The rate increased among women from richer areas to 12.5%, or within the evidence-based range of meeting the need (10–15%) recommended by the World Health Organization (WHO) [25, 26], while staying too low at 5.6% among the poorer group in 2018. ZDHS did not provide information on C-section rates by sector or whether the procedures were elective or emergency.

**Fig. 4:**
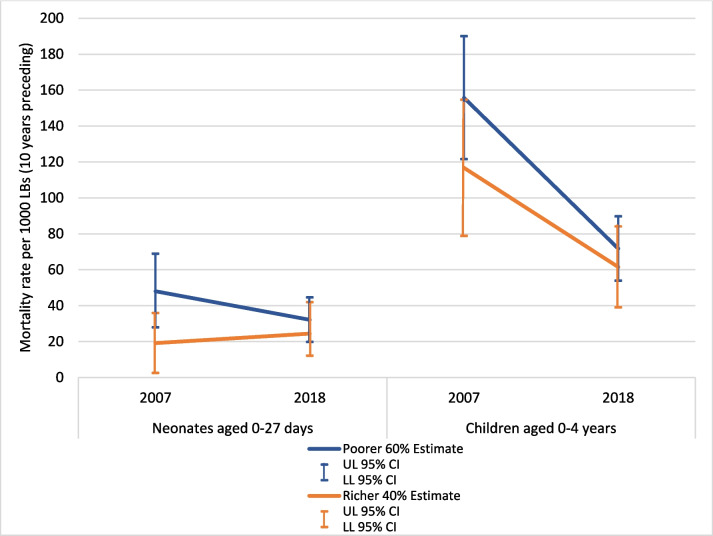
Neonatal and under-5 mortality rates per 1000 live births (average among births in the last ten years) comparing the poorer 60% and richer 40% with confidence intervals (95% CI), ZDHS 2007 and 2018

Neonatal mortality rates appeared to decline both among women from poorer areas and richer ones, with the gap between them closing due to a greater decline among the poorer (Fig. 4). However, confidence intervals were wide, particularly for the richer group (among whom there were fewer births). Declines in deaths among children under age 5 also appeared to be faster among those living in poorer than richer areas, which had more stable estimates. This consistent pattern may indicate a true improvement in the NMR for the poorer group, although the rate was still 7 units higher among the poorer compared with the richer in 2018 (32 versus 25 per 1000 live births, respectively).

Using HMIS data from 2018 to 2021, we examined current MNH service volumes and rates in Lusaka. Government facilities with sufficient HMIS data on MNH services included 34 primary care facilities (urban health centers, posts, or clinics), of which 22 largely served unplanned settlements and 12 primarily planned settlements. Data were used from 12 government hospitals (six first-level hospitals in the unplanned, and two second level, two third level in addition to two police/army hospitals in the planned). Private facilities included 13 primary care facilities or clinics in planned settlements and one in unplanned ones, and 14 hospitals serving the planned settlements.

**Fig. 5:**
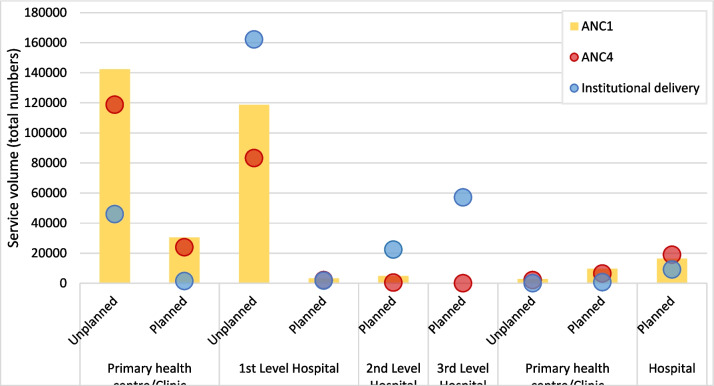
Total (summed) service volumes for ANC1, ANC4, and institutional delivery in public and private facilities by level and catchment area, HMIS 2018 to 2021

The highest numbers of ANC1 visits occurred in public facilities, and particularly those serving unplanned settlements (including primary health centers, clinics, and first-level hospitals) (Fig. 5). Coverage rates of ANC4 to ANC1 were around 70% and 80%, respectively, in facilities serving unplanned settlements. For institutional deliveries, hospitals provided much higher volumes than clinics or primary care facilities, including the hospitals serving unplanned settlements (all first level). The ratio of institutional deliveries to ANC1 visits was higher in hospitals than primary care facilities, as women are often referred to give birth in hospitals even if they attended ANC visits at a primary facility.

As expected, all MNH service volumes were much lower in private facilities (which also mainly served planned settlements). The proportion of ANC4 to ANC1 was just over 1 in private hospitals, and around 80% in primary health centers or clinics. This suggests that many women only attended their later ANC check-ups at hospitals, and went to a lower-level private or any public facility for ANC1. The ratio of institutional deliveries to ANC1 was over 1 in public hospitals, but below 1 at public clinics/primary centers as well as all private facilities, which suggests that more women rely on delivery than antenatal care at public hospitals.

**Fig. 6:**
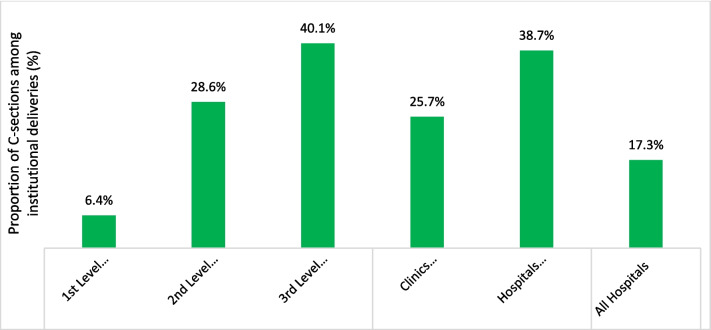
Institutional C-section rates in public and private hospitals or clinics located in unplanned or planned settlements, HMIS 2018 to 2021

Looking at institutional C-section rates between 2018 and 2021 (Fig. 6), rates were lowest in hospitals directly serving unplanned settlements (6.4%). Hospitals located in planned settlements had much higher rates (29% at second level and 40% at third level). Although there are fewer of them, these are larger hospitals conducting the highest volumes of C-sections. Private facilities conducting C-sections were located only in planned settlements. They had lower C-section volumes than the public sector but appeared to have similarly high rates of C-sections as the public second- and third-level hospitals. Primary health centers and public clinics do not provide C-sections.

## Discussion

The urban health advantage appears to be disappearing in many countries. This is due in part to a growing burden to meet the complex needs of more people living in unplanned settlements through health and social services [27]. The results of this study provide a mixed picture of equity trends in MNH in Zambia’s capital city of Lusaka. We found not only notable reductions in coverage gaps for MNH services between 2001 and 2018, but also persistent inequalities (particularly in services with higher intensity or technical quality), which may hinder efforts to reduce inequities in mortality in urban areas [28, 29].

The ZDHS analysis showed noticeable improvements in coverage of any ANC and institutional delivery in the past 20 years. The improvements were slightly greater among the poorer 60% compared to richer 40%, thus narrowing inequality gaps. These findings come in the context of less promising ones, including earlier studies showing that women in urban slum or unplanned settlement communities had widespread challenges accessing services even though well-functioning health facilities are reachable [27]. Our finding that having at least one ANC visit is now nearly universal among the poorest women in Lusaka suggests that ANC availability is good, particularly through the primary health care system and due to removal of user fees since 2006 [30]. However, inequalities have remained in coverage of early and repeated ANC visits, with the poor being disadvantaged. This finding may be explained by informal rules implemented by some health facilities that require male involvement for prevention-of-mother-to-child-transmission interventions, or for women to bring their own supplies, both of which may delay or prevent women from coming to ANC check-ups at facilities near them [12]. To improve timely and repeated ANC visits, services should be inclusive and sensitive to women’s needs regardless of socioeconomic status.

This study also showed improvement compared to previous studies in major inequalities in institutional delivery and skilled birth attendance between rich and poor in Zambia’s urban areas [27, 31]. The narrowing gap in institutional delivery suggests that overall availability and accessibility have improved, as a growing number of health centers and lower-level hospitals provide these services to people living in unplanned settlements. Meanwhile, C-section rates increased enough to meet the recognized general need among women from richer areas (based on analysis of facilities in planned settlements), but remained insufficient to meet the need among poorer groups (based on facilities serving unplanned settlements) [26]. Public first-level hospitals were upgraded recently to provide C-sections in 2018, and user fees were removed. Yet this study suggests that because these facilities have high delivery volumes but low C-section rates, they still refer many emergency cases to second- or third-level hospitals.

Past evidence shows that Lusaka had the highest number of emergency obstetric and newborn care (EmONC) facilities in Zambia, particularly for BEmONC signal function, and relatively good transportation networks and proximity to facilities overall [32–34]. Still, previous facility assessments from 2015 showed an insufficient number of such relative to global recommendations, particularly in the public sector handling most deliveries. Those facilities also were found to be inconsistent over time in regard to having necessary EmONC equipment or capacities [32]. Other assessments found shortages of human resources and management capacity at most public EmONC facilities in Lusaka [14, 32]. Other studies in large urban centers in other countries in sub-Saharan Africa have explored the impact of traffic congestion on delaying women from reaching facilities from home or another facility in emergencies, which may be worth considering in future research as Lusaka’s population continues to grow [35].

This study indicates a need to further improve the capacities of first-level hospitals serving the large number of pregnant women and newborns from unplanned settlements, by ensuring enough adequately trained personnel, equipment, and supplies at all times. Strengthening referral systems would also be valuable when needed, including by ensuring availability of ambulances and trained drivers per zone as per the referral policies, and potentially by integrating them more through digitized mechanisms [24]. Future research should investigate the extent to which urban inequalities in MNH care are caused by access issues (such as inadequate birth preparedness and danger sign recognition, indirect costs, insecurity at night, or insufficient transport), compared to readiness and quality issues (such as too few skilled attendants and especially specialists, poor experiences of care, or limited blood and other supplies) [12, 27, 36, 37].

This study had some limitations. The ZDHS GIS coordinate scrambling caused challenges in identifying poorer and richer households by geographic location; we assessed the correlation and best wealth cutoffs using toilet characteristics as a key indicator in this context. The small ZDHS sample of the Lusaka urban cluster made it difficult to maintain robust estimates if we disaggregated wealth asset scores further. For neonatal mortality rates, ZDHS had large standard errors, and HMIS had insufficient reporting and was excluded. MMR comparisons also could not be included. The association of inequalities in coverage and the NMR would have been valuable to assess, but the differential recall periods for these indicators (a couple years for coverage, 10 years for the NMR due to low numbers) made it hard to do so meaningfully.

We did not compare HMIS results over time due to data limitations before 2018. Moreover, HMIS provides volumes and not coverage estimates because of the lack of reasonable denominator estimates at this granular level. Neither ZDHS nor HMIS has data to distinguish elective from emergency C-sections. Women using facilities in planned settlements could also live in unplanned settlements and voluntarily bypass facilities closest to them, though this requires a one-off fee. Private facilities provided a minority of MNH services availed, but the lack of HMIS data from them likely led to underestimated total volumes. The COVID-19 pandemic may have also impacted the observed total volumes, although we conducted separate analyses of the HMIS volumes in 2020 versus earlier years and found that the reductions were not as large within urban as rural areas [38]. As HMIS data can be of variable quality, we conducted facility-wise data quality assessment of completeness and outliers and adjusted for missing values. We did not notice outliers so large or frequent as to likely affect the total volumes and thus did not adjust for them, though this could be a limitation.

Notwithstanding the limitations, the complementary nature of the datasets is a strength worth noting. For instance, while HMIS data lacked information on individual characteristics for equity analysis, they show where services are available and their volumes. Also, while the DHS data did not specify where services are available or their volumes, they contain individual and household characteristics of service users to allow equity analysis. Integrating these datasets with population estimates and health facility location and characteristics provides new insights previously unseen in Lusaka, and could be a worthwhile approach to future research in cities within the region.

## Conclusions

Notable improvements in coverage of any ANC and institutional delivery services have been achieved, thereby narrowing the gaps between poorer families in unplanned settlements and others in Lusaka City, Zambia. Ongoing inequalities in quality ANC, emergency delivery care, and ultimately birth outcomes could be better addressed by ensuring strong outreach to all pregnant women in unplanned settlements, to heighten access to timely and continuous ANC services, and to augment capacities to provide EmONC services and responsive referral systems at facilities serving them. Moving beyond improving equity in contact with services towards ensuring high-quality and timely services could be the next step towards responsive approaches that improve the health and well-being of all women and their children in Lusaka.

## Supplementary Information

Below is the link to the electronic supplementary material.

Supplementary file1 (DOCX 25 KB)

## Data Availability

Data is available publicly online upon request.
