# Supplementary material for: Trends and Inequalities in Maternal and Newborn Health Services for Unplanned Settlements of Lusaka City, Zambia
Source: J Urban Health. 2024 Mar 8;101(Suppl 1):125–37. doi: 10.1007/s11524-024-00837-z (PMC11602902; doi:10.1007/s11524-024-00837-z)
Supplement: Supplementary file 1 — Supplementary file1 (DOCX 25 KB) [file 11524_2024_837_MOESM1_ESM.docx]

**Supplementary Material**

**Supplementary Figure 1. Intervention coverage (%) among births in the last five years by household toilet type/sharing versus wealth quintiles (60% poorer vs. 40% richer using DHS asset indices) in urban Lusaka, ZDHS 2018**

**Supplementary Table 1. Maternal and newborn health intervention coverage (with 95% confidence intervals) among births in the last five years in urban Lusaka, ZDHS 2001–2018**

| **Proportion (%) of births in last five years** | | **2001** | | **2007** | | **2013** | | **2018** | |
| --- | --- | --- | --- | --- | --- | --- | --- | --- | --- |
|  |  | **Estimate** | **95% CI** | **Estimate** | **95% CI** | **Estimate** | **95% CI** | **Estimate** | **95% CI** |
| **ANC 1+** | **Poorer 60%** | 97.8 | 95.2-99.0 | 100.0 | NA | 98.9 | 97.9-99.4 | 97.8 | 96.3-98.7 |
|  | **Richer 40%** | 98.1 | 95.0-99.3 | 100.0 | NA | 100.0 | NA | 100.0 | NA |
| **Early ANC** | **Poorer 60%** | 16.8 | 12.9-21.7 | 20.9 | 16.3-26.4 | 20.6 | 18.0-23.5 | 25.2 | 21.9-28.8 |
|  | **Richer 40%** | 29.1 | 23.2-35.8 | 29.5 | 23.1-36.7 | 27.8 | 24.1-31.9 | 32.7 | 28.3-37.4 |
| **ANC 4+** | **Poorer 60%** | 82.7 | 77.8-86.6 | 52.2 | 45.9-58.3 | 45.3 | 41.9-48.7 | 50.9 | 46.9-54.8 |
|  | **Richer 40%** | 86.3 | 80.9-90.4 | 59.8 | 52.3-66.9 | 66.0 | 61.8-70.0 | 67.1 | 62.4-71.5 |
| **Institutional delivery** | **Poorer 60%** | 77.9 | 73.5-81.7 | 84.0 | 79.8-87.5 | 88.2 | 86.1-90.0 | 91.2 | 89.0-93.0 |
|  | **Richer 40%** | 87.2 | 82.5-90.8 | 93.4 | 89.4-96.0 | 98.3 | 97.0-99.1 | 97.1 | 95.1-98.2 |
| **C-sections** | **Poorer 60%** | 4.8 | 3.0-7.3 | 4.8 | 3.0-7.5 | 4.7 | 3.6-6.2 | 5.6 | 4.2-7.5 |
|  | **Richer 40%** | 4.9 | 2.9-8.4 | 11.9 | 8.3-16.8 | 11.5 | 9.3-14.2 | 12.5 | 9.9-15.7 |
